# Edible Tubers as a Source of Bioactive Compounds in Baked Goods: Benefits and Drawbacks

**DOI:** 10.3390/molecules30132838

**Published:** 2025-07-02

**Authors:** Rafał Wiśniewski, Ewa Pejcz, Joanna Harasym

**Affiliations:** 1 Department of Biotechnology and Food Analysis, Wroclaw University of Economics and Business, Komandorska 118/120, 53-345 Wroclaw, Poland; rafal.wisniewski@ue.wroc.pl (R.W.); joanna.harasym@ue.wroc.pl (J.H.); 2 Adaptive Food Systems Accelerator-Science Centre, Wroclaw University of Economics and Business, 53-345 Wroclaw, Poland

**Keywords:** edible tubers, bioactive compounds, polyphenols, antioxidants, dietary fiber, prebiotics, functional bakery, acrylamide, FODMAPs, cyanogenic glycosides

## Abstract

Root and tuber vegetables—such as beetroot (*Beta vulgaris*), carrot (*Daucus carota*), cassava (*Manihot esculenta*), potato (*Solanum tuberosum*), taro (*Colocasia esculenta*), and Jerusalem artichoke (*Helianthus tuberosus*)—are increasingly recognized not only for their nutritional value but also for their richness in bioactive compounds, including polyphenols, dietary fiber, resistant starch, and prebiotic carbohydrates that exhibit varying levels of antioxidant, anti-inflammatory, and glycemic-regulating properties. Incorporating these vegetables into baked goods offers both functional and technological benefits, such as improved moisture retention, reduced acrylamide formation, and suitability for gluten-free formulations. The processing conditions can significantly influence the stability and bioavailability of these bioactive components, while the presence of antinutritional factors—such as phytates, cyanogenic glycosides, and FODMAPs (fermentable oligo-, di-, monosaccharides, and polyols)—needs careful optimization. The structured narrative literature review approach allowed collecting studies that examine both the beneficial and potential drawbacks of tuber-based ingredients. This review provides a comprehensive overview of the chemical composition, health-promoting effects, and technological roles of edible tubers in bakery applications, also addressing current challenges related to processing, formulation, and consumer acceptance. Special emphasis is placed on the valorization of tuber by-products, enhancement of functional properties, and the promotion of sustainable food systems using zero-waste strategies.

## 1. Introduction

**Figure 1:**
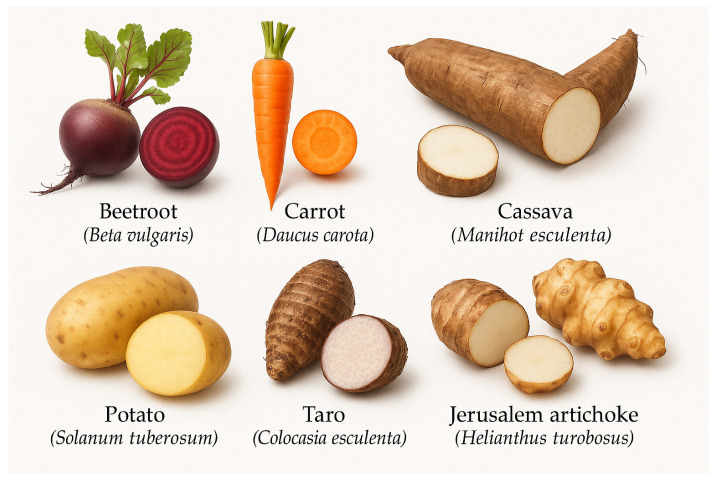
Edible tubers.

Edible tubers—including beetroot (*Beta vulgaris*), carrot (*Daucus carota*), cassava (*Manihot esculenta*), potato (*Solanum tuberosum*), sweet potato (*Ipomoea batatas*), yam (*Dioscorea* spp.), Jerusalem artichoke (*Helianthus tuberosus*), taro (*Colocasia esculenta*), and yacon (*Smallanthus sonchifolius*)—represent a nutritionally and functionally diverse group of crops with growing importance in health-oriented food systems (Figure 1). These tubers are increasingly investigated for their potential as ingredients in bakery products due to their content of bioactive compounds such as phenolic acids, flavonoids, carotenoids, anthocyanins, dietary fiber, resistant starch, and prebiotic oligosaccharides. In addition to enhancing nutritional profiles, these crops offer technological advantages such as improved texture, natural coloring, moisture retention, and gluten-free applicability. However, their use also requires consideration of antinutritional compounds, including phytates, oxalates, and cyanogenic glycosides, as well as FODMAP (fermentable oligo-, di-, monosaccharides, and polyols) content, which may impact consumer tolerance [1,2]. This review synthesizes current knowledge on the compositional, functional, and health-promoting attributes of these tubers in the context of their utilization in baked goods.

The incorporation of tuber flours and extracts into bakery recipes aligns with evolving consumer preferences toward clean-label formulations, plant-based diets, and nutritionally enriched foods [1,2]. These crops exhibit unique functional and rheological properties, including high water-binding capacity, improved crumb structure, and natural sweetness [3]. Additionally, their lack of prolamins facilitates gluten-free product development for individuals with celiac disease or non-celiac gluten sensitivity [4]. Despite many advantages, tubers also present several important challenges. Antinutritional factors such as phytates, oxalates, and cyanogenic glycosides can limit their utilization, while the high FODMAP content may affect some consumers’ tolerance. Additionally, processing techniques alter the stability and bioavailability of the bioactive compounds in some tubers, leading to potential nutritional loss.

Nutritionally, tubers are rich in a diverse array of bioactives. Purple sweet potatoes are abundant in anthocyanins such as peonidin-3-glucoside, which modulate Nrf2 and NF-κB pathways and exhibit hepatoprotective and neuroprotective activity [3]. Jerusalem artichoke is a major source of inulin-type fructans, providing not only dietary fiber but also serving as a selective substrate for gut microbiota, promoting the growth of *Bifidobacterium* and *Lactobacillus* spp. [3]. Likewise, Chinese yam contributes unique polysaccharides, resistant starches, steroidal saponins, and mucilage fibers with proven anti-diabetic, hypocholesterolemic, and immunomodulatory properties [1,5]. A growing body of evidence also supports the valorization of tuber processing by-products. Peels, pomace, and after-extraction residues are increasingly explored as sources of concentrated antioxidants, fibers, and micronutrients, with viable applications in fortifying bakery goods while supporting zero-waste and circular bioeconomy strategies [2,6].

Although considerable progress has been made, the scientific literature remains fragmented. Most available studies are focused on specific species or narrow nutritional attributes (e.g., glycemic response or fiber enrichment), without a comprehensive synthesis across multiple types or technological applications [7]. This gap inhibits the optimized utilization of tubers in bakery systems, especially in the context of bioactive retention, process optimization, and health outcomes.

Therefore, this review aims to provide an integrative synthesis of the biochemical, prebiotic, technological, and health-relevant attributes of selected edible tuber crops. A particular focus is placed on their applicability in the formulation of functional bakery products, including the valorization of by-products, compositional diversity, and morphological suitability for processing (Table 1).

## 2. Methods

A structured narrative literature review was conducted without adherence to PRISMA guidelines, as the aim was to provide a broad and integrative synthesis of existing knowledge rather than a narrowly focused quantitative analysis. This approach allowed for the inclusion of diverse sources and research designs, which would not have been feasible through a conventional systematic review. The search encompassed peer-reviewed publications up to April 2025, focusing on the biochemical, functional, technological, and nutritional properties of edible root and tuber crops used in baked goods. Specific keywords included “root vegetables”, “tubers”, “cassava”, “sweet potato”, “potato”, “taro”, “yam”, “beetroot”, “Jerusalem artichoke”, “inulin”, “resistant starch”, “polyphenols”, “antioxidants”, “dietary fiber”, “glycemic index”, “acrylamide”, “functional bakery products”, and “processing effects.” Reference lists of all retrieved articles and prior reviews were manually screened to identify additional relevant studies. Only studies published in English were included due to practical limitations related to translation and consistency. This review did not include a formal risk of bias assessment or meta-analysis and should be interpreted as a qualitative synthesis of current literature.

### 2.1. Eligibility Criteria

Included studies were original peer-reviewed articles published in English that focused on the biochemical, nutritional, technological, or functional characteristics of root and tuber crops used in baked food products. Reviews, conference abstracts, non-peer-reviewed literature, and studies unrelated to food applications were excluded. Only studies involving human food uses (not feed, fuel, or non-edible uses) were considered. The language restriction to English was due to resource limitations for translation and consistency in interpretation.

### 2.2. Study Selection

After removing duplicates, the titles and abstracts of the retrieved records from PubMed, Scopus, and Web of Science were screened for relevance. Full texts of potentially eligible studies were then evaluated according to predefined inclusion criteria. Additionally, reference lists from included articles and relevant reviews were manually screened to identify further eligible sources. Two authors performed the selection process independently, and disagreements were resolved through discussion. As this was a structured narrative review, no formal quality appraisal or risk of bias assessment was conducted. In total, 193 articles were included in the final synthesis.

### 2.3. Data Extraction and Synthesis

Key data from included studies were extracted into a structured summary table, covering crop type, functional component(s), processing methods, and observed nutritional or technological outcomes. The results were synthesized narratively, highlighting common findings, emerging trends, and differences across crop types and processing techniques. No meta-analysis or formal statistical synthesis was conducted due to the heterogeneity of study designs and outcomes.

## 3. Bioactive Compounds in Plant Tubers

Root and tuber vegetables—including potato (*Solanum tuberosum*), carrot (*Daucus carota*), beetroot (*Beta vulgaris*), cassava (*Manihot esculenta*), taro (*Colocasia esculenta*), and Jerusalem artichoke (*Helianthus tuberosus*)—are rich in bioactive compounds such as polyphenols, resistant starch, vitamins, minerals, and natural pigments. These compounds exert antioxidant, anti-inflammatory, glycemia-modulating, and gut-supporting effects and are increasingly applied in the development of functional bakery products. Their bioactivity is strongly affected by species, genotype, environmental conditions, and processing techniques like baking, boiling, or drying, which may enhance or degrade specific phytochemicals [15,16,29,30,31].

### 3.1. Polyphenolic Compounds

Polyphenols, particularly phenolic acids and flavonoids, are major contributors to the antioxidant and health-promoting potential of root vegetables. Among phenolic acids, hydroxycinnamic acids such as ferulic, caffeic, p-coumaric, and chlorogenic acid dominate. Ferulic acid is found at high levels in carrots, taro, and beetroot; it is known for strong free radical scavenging and is especially abundant in outer tissues [32,33,34,35]. Chlorogenic acid is the principal phenolic in potatoes, especially in pigmented and peel-rich cultivars, comprising up to 90% of the total phenolics and reaching 200 mg gallic acid equivalent (GAE)/100 g dry weight (dw) [32,33,34,35,36,37]. Cassava peels and stems are rich in hydroxybenzoic acids like gallic and protocatechuic acid, contributing to moderate antioxidant activity [32,33,34,36,38].

Flavonoids such as rutin, kaempferol, quercetin, and luteolin occur in various tubers, often in lower concentrations than phenolic acids, but with synergistic effects. Rutin is prominent in carrots and potatoes, while quercetin glycosides are well documented in purple-flesh potato cultivars [32,33,37,38,39,40,41,42]. Jerusalem artichoke offers a unique flavonoid profile, including methoxylated compounds like hymenoxin and liquiritigenin, which have been linked to estrogenic, antioxidant, and anti-inflammatory properties [43,44].

The total phenolic content varies significantly between crops. Beetroot contains the highest levels (352.46–489.06 mg GAE/100 g DW), with white varieties dominated by ferulic acid and red varieties enriched in both caffeic acid and betalains, which provide both color and biological activity [33,35,39,40,45]. Carrots contain 10–40 mg GAE/100 g fresh weight (fw), with black cultivars showing up to ninefold higher content than orange types, highlighting cultivar-driven variability [33,37,46]. Cassava extracts from stems contain p-coumaric acid, scopoletin, and syringaldehyde, with antioxidant effects concentrated in ethyl acetate fractions [38].

In potatoes, phenolic content and composition are strongly genotype dependent. Pigmented varieties such as ‘Vitelotte’ exhibit high anthocyanin levels (up to 54.09 mg/100 g FW), along with chlorogenic, caffeic, p-coumaric acids, and flavonoids such as quercetin-3-O-rutinoside [41,42]. In Jerusalem artichoke, ultra-high performance liquid chromatography coupled with electrospray ionization tandem mass spectrometry (UHPLC-ESI-MS/MS) analysis identified over 50 polyphenolic compounds, including chlorogenic, neochlorogenic, and feruloylquinic acids, present predominantly as glycosylated derivatives, enhancing their solubility and bioactivity [43,44].

Importantly, processing modulates polyphenol availability and functionality. Baking at 200 °C enhances the extractability of chlorogenic acid in potatoes and ferulic acid in beetroot, while freeze-drying concentrates phenolics in carrot and cassava powders [15,16,29,30,31,35,39,40,47]. Fermentation can increase the proportion of free phenolic acids, as shown in beetroot, enhancing both antioxidant and angiotensin-converting enzyme (ACE)-inhibitory activity [35,45].

In the context of bakery applications, polyphenols serve not only as antioxidants but also as natural preservatives and pigment stabilizers. Their presence in root vegetable flours improves nutritional value and extends the shelf life of functional breads and snacks. However, their bioavailability and sensory effects are sensitive to thermal treatment and matrix interactions, requiring optimized formulation and processing.

### 3.2. Resistant Starch

Resistant starch (RS), a form of dietary fiber, escapes enzymatic digestion in the small intestine and undergoes fermentation in the colon. It promotes gut health by stimulating beneficial microbiota (e.g., Bifidobacteria, Lactobacilli), enhances short-chain fatty acid (SCFA) production—particularly butyrate and propionate—and exhibits anti-inflammatory, anticarcinogenic, and cholesterol-lowering effects [48,49,50,51,52,53]. RS also improves glycemic control, reduces postprandial glucose spikes, and supports weight management by promoting satiety [50,53].

RS is categorized into five types (RS1–RS5) based on structure and digestibility: RS1 (physically inaccessible), RS2 (native granular starch, e.g., raw potato), RS3 (retrograded starch from cooled cooked starch), RS4 (chemically modified), and RS5 (amylose–lipid complexes) [49,53,54].

Root vegetables, particularly potatoes, taro, cassava, and yams, are natural sources of RS—unlike refined cereal products. RS content varies by species, cultivar, and processing conditions. Raw potatoes contain up to 47–59% RS in dry matter [55], and potato flour retains 36–40% RS, depending on starch structure (crumbly > waxy types) [56,57]. RS content in lesser yam reaches 23.3%, while cassava roots show variability between 5.0 and 19.6%, depending on the cultivar and drying method [58,59]. Taro presents notable regional differences, with RS levels ranging from 15.1% to over 20.8%, and these levels can be further enhanced (~30%) by cold plasma treatment, a promising pre-processing method for food applications. In contrast, sweet potatoes exhibit low RS content (~3.2%), limiting their prebiotic potential unless modified [58].

From a technological standpoint, RS is particularly relevant in baking. Retrogradation during cooling of baked starches (RS3) enhances RS formation, enabling functional breads with improved glycemic index (GI) profiles. Potato and cassava flours, for instance, can enrich bakery formulations with RS, provided the baking and cooling stages are optimized to retain retrograded starch fractions. Moreover, RS’s water-binding capacity may influence dough rheology and texture, necessitating formulation adjustments in gluten-free applications. RS from root and tuber crops contributes not only to metabolic health but also offers functional potential in baking, especially in reformulated or high-fiber bread products. Their inclusion supports both nutrition-focused innovation and textural diversification in modern bakery systems.

### 3.3. Vitamins and Minerals

Root and tuber vegetables are important dietary sources of essential vitamins and minerals, contributing to immune function, antioxidant defense, cardiovascular protection, and metabolic regulation. The content of micronutrients varies by species, cultivar, and processing methods, with notable examples including *Beta vulgaris* (beetroot), *Daucus carota* (carrot), *Manihot esculenta* (cassava), *Solanum tuberosum* (potato), *Colocasia esculenta* (taro), and *Helianthus tuberosus* (Jerusalem artichoke) [30,32,33].

Vitamin A, particularly in the form of β-carotene, is abundant in orange-fleshed root vegetables like carrots, reaching levels between 6000 and 54,800 µg/100 g fresh weight, and representing up to 80% of their total carotenoids [32]. Other carotenoids such as α-carotene, lutein, and zeaxanthin enhance antioxidant potential, particularly in yellow and purple cultivars [32,37]. These compounds not only support visual and immune health but also serve as precursors for vitamin A in functional baked goods enriched with vegetable powders.

Vitamin C, a potent antioxidant and cofactor for collagen synthesis and immune regulation, is present in moderate amounts in most root crops—e.g., beetroot (up to 4.9 mg/100 g), Jerusalem artichoke (up to 14 mg/100 g), and potatoes (up to 30 mg/100 g) [33,35,47,60]. Vitamin C is partially degraded during high-temperature baking, although its retention can be improved through vacuum drying or the use of pre-gelatinized flours [32,47].

B-complex vitamins, including thiamine (B1), riboflavin (B2), niacin (B3), and pyridoxine (B6), are distributed across carrots, cassava leaves, potatoes, taro, and Jerusalem artichoke [32,60,61,62,63]. These vitamins are vital for energy metabolism and nervous system function, and their incorporation via tuber flours can help reduce deficiencies in refined baked products.

Among minerals, potassium (K) is most abundant across root vegetables, contributing to electrolyte balance and muscle function. Taro (2271–4276 mg/100 g), parsley (up to 3375 mg/kg), carrots (~1524 mg/kg), and Jerusalem artichoke (~490 mg/100 g) all provide significant potassium content [32,47,63]. Calcium, essential for bone metabolism, ranges from ~15–35 mg/100 g in cassava to over 300 mg/kg in celery and parsley roots [32].

Magnesium, required for enzymatic reactions and cardiovascular health, appears in levels up to 543 mg/100 g in taro and ~30–80 mg/100 g in cassava [47,60,62]. Other essential trace elements include iron (notably high in taro and parsley), zinc, copper, and manganese, found in varying amounts across all studied tubers [32,47,63]. Beetroot and Jerusalem artichoke also contribute appreciable levels of folate, which supports hematopoiesis and fetal development [35,60].

Nutritionally, the regular intake of 200 g of raw carrots or parsley can provide over 20% of daily needs for iron, manganese, or copper, highlighting the potential of root vegetables to combat micronutrient deficiencies [32,47]. However, culinary processing affects vitamin and mineral retention. For example, β-carotene bioavailability increases with heat-induced cell wall disruption, while vitamin C losses can be minimized using steaming or vacuum-drying techniques [32].

From a technological and nutritional perspective, incorporating tuber flours into bakery products (e.g., cassava, taro, beetroot) allows for micronutrient enrichment, especially in gluten-free or reformulated products. Root vegetables serve not only as bulk or textural agents but also as natural carriers of vitamins and minerals with functional relevance. Moreover, the color and antioxidant contribution of carotenoid- and betalain-rich vegetables (e.g., carrot, beetroot) can enhance the visual appeal and health perception of baked goods.

In summary, root vegetables offer a broad spectrum of micronutrients, including vitamins A, C, and B-complex as well as potassium, magnesium, calcium, and iron. Their integration into bakery matrices can support improved nutritional profiles, especially when using minimally processed or thermally optimized flours [32,35,37,40,47,60,61,62,63].

### 3.4. Pigments

Root vegetables accumulate a wide range of bioactive pigments—including betalains, carotenoids, and anthocyanins—that contribute not only to their vivid coloration but also to their health-promoting potential. These pigments differ chemically and functionally, and their distribution varies across tuber species, with beetroot, carrot, purple potato, and cassava being especially rich sources [29,30,31,32,33,34,35,39,40,47].

Betalains, unique to species such as beetroot (*Beta vulgaris*), consist of betacyanins (e.g., betanin, isobetanin) and betaxanthins (e.g., vulgaxanthin I and II), which are synthesized from tyrosine and exhibit antioxidant, anti-inflammatory, and anticancer activities. Their concentrations can reach 5.35–7.89 mg/g (betacyanins) and 3.81–5.51 mg/g (betaxanthins) dw. Due to their strong chromatic properties and radical-scavenging capacity, they are widely used as natural food colorants in bakery glazes and fillings. However, they are heat sensitive; optimal stability is retained through freeze-drying or low-temperature dehydration (~70 °C) [30,31,32,33,35].

Carotenoids, fat-soluble pigments responsible for yellow to orange hues, are abundant in carrots (*Daucus carota*) but also present in sweet potatoes, cassava, and taro. The most common is β-carotene, often accounting for up to 80% of the total carotenoids in carrots (6000–54,800 µg/100 g fw) [32]. Other important carotenoids include α-carotene, lutein, and zeaxanthin, which act as antioxidants and vitamin A precursors. Importantly, processing methods such as steam blanching and vacuum drying improve bioavailability via trans-to-cis isomerization, enhancing the functionality of carotenoid-rich tuber flours in baked applications [32,33,47].

Anthocyanins, water-soluble flavonoid pigments, impart purple and blue hues and are concentrated in purple-fleshed potatoes and cassava. Major anthocyanins such as cyanidin-3-glucoside and delphinidin derivatives contribute to the high antioxidant and anti-inflammatory potential of these cultivars [11,15,22]. Their content in purple potatoes can range from 2.5 to 8.0 mg/100 g fw, varying by genotype and environmental conditions. While anthocyanins are moderately heat sensitive, boiling and steaming offer acceptable retention levels, making them viable for functional bread and pastry coloration [29,30,31,32,33,34,35,39,40,47].

The bioactive pigment profile of root vegetables is both diverse and functionally relevant. Betalains (e.g., in beetroot) offer antioxidant colorant potential. Carotenoids (e.g., in carrots and taro) support visual and immune health. Anthocyanins (e.g., in purple potatoes and cassava) provide cardiovascular and cognitive protection. Their incorporation into bakery products, whether through powders, purées, or flours, can enhance both the nutritional value and sensory attributes of final goods. Applying gentle processing methods ensures optimal retention of these sensitive pigments, facilitating the development of naturally colored, health-promoting bakery formulations.

## 4. Health Benefits of Tuber

### 4.1. Antioxidant Effect

Root and tuber vegetables are rich in antioxidants, particularly polyphenolic compounds, carotenoids, betalains, vitamins (e.g., C, E, A), and essential minerals. These bioactive constituents play a vital role in neutralizing free radicals, reducing oxidative stress, and supporting the prevention of chronic diseases such as cardiovascular disorders, diabetes, and cancer [33,35,64,65,66,67,68,69] (Table 2).

The antioxidant activity of these crops is typically assessed using DPPH (2,2-diphenyl-1-picrylhydrazyl), ABTS (2,2′-azino-bis(3-ethylbenzothiazoline-6-sulfonic acid)), FRAP (ferric reducing antioxidant power), and ORAC (oxygen radical absorbance capacity) assays. For instance, beetroot exhibits high antioxidant capacity (DPPH: 0.45–1.30 mmol trolox equivalent antioxidant capacity (TEAC)/100 g; ABTS: 0.90–1.80 mmol TEAC/100 g), mainly due to betalains (128.7–797 mg/100 g) and phenolic acids (e.g., gallic, caffeic, ferulic acids) [16,33,64,70,71]. Carrots, especially purple ones, contain hydroxycinnamic acids (e.g., chlorogenic, ferulic) and anthocyanins contributing to DPPH values of 56–90 mg GAE/100 g [33,62,68,72,73,74].

Sweet potatoes show antioxidant activity up to 25.8 µmol TEAC/g, particularly in purple-fleshed varieties due to anthocyanins, while orange-fleshed types are rich in β-carotene (up to 131 µg/g) [13,64,65,66,67,68,69,75,76]. Cassava and taro possess moderate antioxidant activity enhanced by fermentation or thermal processing, which increases polyphenol bioavailability [22,23,73,77,78,79,80,81,82,83,84,85,86,87]. Yam, potato, and Jerusalem artichoke also demonstrate significant antioxidant properties due to their diverse phenolic profiles [11,47,88,89,90,91,92,93,94,95,96,97,98].

**Table 2:** Phytochemical composition and health effects of edible roots and tubers.

Vegetables	Compounds Type	Compounds Class	Compound Average Content	Health Benefits	References
Carrot	Polyphenolic compounds	Caffeic acid; ferulic acid; sinapic acid; vanillic acid; *p*-coumaric acid; p-hydroxybenxoic acid; and total phenolic	24 mg/100 mg fw; 2.4 mg/100 g fw; 0.06 mg/100 g fw; 1.2 mg/100 g fw; 0.71 mg/100 g fw; 4.2 mg/100 g fw; and 7.3–224 mg/100 g fw	Antioxidant, anti-inflammatory, antidiabetic and anticancer	[32,68,72,73,74]
	Carotenoids	β-Carotene; α-carotene; lutein (orange, purple, red); lycopene (red); zeaxanthin; β-cryptoxanthin; and total of carotenoids	0.09 to 7.6 μg/g dry kernel, 8.2 mg/100 g fw root; 3.5 mg/100 g fw root; 0.1 to 28 μg/g dry kernel, 250 μg μg/100 g fw root; 1 μg/100 g fw root; 0.01 to 8.1 μg/g dry kernel; 0.08 to 2.45 μg/g dry kernel; and 6–54.8 mg/100 g fw root		
	Vitamins	Vitamin A/provitamin A (β-carotene); vitamin E (tocopherols); vitamin C	700 μg; 0.19–15 mg ^1^; 1–5.3 mg/100 g fw		
	Macro and micro elements	K; Ca; Mg; Mn Fe; Zn; Cu	152.4 mg; 31.4 mg; 27.2 mg; 0.58 mg; 0.25 mg; 0.32 mg; 0.07 mg/100 g fw		
Beetroot	Polyphenolic compounds	Gallic acid; chlorogenic acid; caffeic acid; ferulic acid; myricetin; luteolin; quercetin; epicatechin; and total phenols	36.40–65.93 mg; 1.7–4.67 mg; 0.74–0.90 mg; 0.54–1.71 mg; 0.27–0.30 mg; 0.13–0.14 mg; 0.1–0.13 mg; 3.20 mg/100 g fw; and 245 mg GAE/100 g fw ^3^	Antioxidation, anti-inflammatory, anti-hypertensive, decrease of oxidative stress and inflammation in animals studies	[16,71,72]
	Betalains	Total betalains (isobetanin, 2,17′-bidecarboxy-neobetanin, miraxanthin II, vulgxanthin I (deep read), Vulgaxanthin I, indicaxanthin, miraxanthin (yellow)); betalain	370 mg/100 g; 128.7–797 mg/100 g		
	Carotenoids	a-Carotene; lycopene	22 mg/100 g fw; 0.03 mg/100 g fw		
	Vitamins	Vitamin B; vitamin A; vitamin C; riboflavin; vitamin B6; folacin; niacin	0.3–0.4 mg ^2^; 36 IU; 4.9 mg; 0.04 mg; 0.067 mg; 109 mcg; 0.334 mg/100 g fw		
	Macro and micro elements	Potassium; sodium; phosphorus; calcium; magnesium; iron; zinc; copper; manganese; selenium	325 mg; 78 mg; 40 mg; 16 mg; 23 mg; 0.80 mg; 0.35 mg; 0.075 mg/100 g; 0.33 mg/100 g; 0.7 mg/100 g		
Taro	Polyphenolic compounds	Total catechin; trans-ferulic acid; anthocyanins quercetin	35.5 mg; 26.80 mg/100 g de; 16 mg/100 g corm skin; 2.9 mg/100 g fw	Antimetastatic, antioxidant, anticancer, anti-inflammatory, antidiabetic, antimicrobial	[77,78,79,80,81,82,83,84,85,86]
	Carotenoids	β-Carotene	10.4–18.5 mg/100 g taro flour		
	Vitamins	Vitamin A; vitamin C; vitamin E; thiamin; riboflavin; niacin; vitamin K	8.92 mg; 4.5–10.29 mg; 1.89–2.38 mg; 0.21 mg; 0.02–0.04 mg; 0.58 mg/100 g dw, 0.001 mg/100 g		
	Macro and micro elements	Calcium; iron; magnesium; phosphorus; sodium; potassium; manganese; zinc; copper	41–782.15 mg; 1.16–218.50 mg; 7.3–543.90 mg; 1.39 mg; 6.2–25.6 mg; 224–372.40 mg; 0.13–221.30 mg; 5.14–392.23 mg; 0.67–231.70 mg		
Sweet potato	Polyphenolic compounds	Chlorogenic acid; neochlorogenic acid	56.3 mg, 620–2024 mg (peel), 88–252 mg (flesh); 5.18 mg, 53–83 mg/kg dw (peel)	Antioxidant, anti-inflammatory, hypoglycemic anticancer, hepatoprotective	[13,68,73,75,76]
	Carotenoids	β-Carotene (orange)	<1 (white cultivars)–131 μg/g fresh roots (orange cultivars)		
	Vitamins	Vitamin C; thiamin; niacin; vitamin B6; vitamin K	14.8 mg; 0.045 mg; 0.43 mg; 0.12; 0.2 μg/100 g fw		
	Macro and micro elements	Sodium; potassium; calcium; magnesium; iron; zinc; manganese; copper	71.1 mg; 1134 mg; 60.7 mg; 31.3 mg; 1.4 mg; 1.1 mg; 0.5 mg; 0.5 mg/100 g		
Yam	Polyphenolic compounds	Total anthocyanin; sinapic acid, ferulic acid; total polyphenols	31 mg; 131 mg; 31.3 mg/100 g dm; 76.64–324.69 μg GAE/g fw ^3^	Antioxidant, anti-inflammatory, antimicrobial, anticancer, antidiabetic	[20,73,88,94,95]
	Carotenoids	All-trans-β-carotene; β-carotene epoxides; provitamin A	96–332 μg; 96–1670 μg; 102–927 μg/100 g dw		
	Vitamins	Vitamin C; vitamin E	82.13 mg; 88.33 mg/100 g fw		
	Macro and micro elements	Sodium; magnesium; calcium; iron; zinc; copper; cobalt; manganese	11.4–12.06 mg; 7.11–566 mg; 21.8–660 mg; 2.05 mg; 7.6–14.1 mg; 0.5 mg; 0.57–0.82 mg; 0.26–10.6 mg/100 g fw		
Cassava	Polyphenolic compounds	Gallic acid; chlorogenic acid; ferulic acid; rutin; caffeic acid; catechin	144.2 μg; 65.9 μg; 118.8 μg; 815 μg; 13.8; 12.8 μg/mL ethanolic extract of cassava	Antioxidant, anti-inflammatory, anticancer, antimicrobial, hypoglycemic	[22,23,73,87]
	Carotenoids	β-Carotene (orange color and cream color), 9-Z- β-carotene, 13-Z- β-carotene (orange color)	0.2 to 4.9 μg/g fresh roots		
	Vitamins	Vitamin A; niacin; riboflavin; thiamin; vitamin C	0.005–0.04; 0.6–1 mg; 0.03–0.06 mg; 0.03–0.28 mg; 14.9–50 mg/100 g fw		
	Minerals	Calcium; magnesium; phosphorus; iron; potassium; copper; manganese; sulfur; sodium; zinc	16–176 mg; 30–80 mg; 6–152 mg; 0.3–14 mg; 250–720 mg; 0.3–0.6 mg; 273 ppm; 7.6–21.3 mg; 1.4–4.1 mg/100 g fw		
Potatoes	Polyphenols	Chlorogenic acid; cryptochlorogenic acid; neochlorogenic acid; caffeic acid; caffeoyl putrescine; rutinose; kaempferol-3-rutinose; anthocyanins content	21.9–80.4 mg ^4^; 1.0–12.6 mg ^5^; 0.1–2.9 mg ^4^; 0.5–5.2 mg ^6^; 0.2–1.3 mg ^4^; 0.29–1.36 mg ^7^; 0.13–0.46 mg ^8^; 5.5–368 mg/100 g fw ^9^	Free radical, oxyradical, hydroxyl radical scavenging activity, protection of liver injury (rats), cholesterol-lowering effects in rats	[11,89,90,91]
	Carotenoids	Carotenoids content in flesh	50 to 2000 μg/100 g fw ^10^		
	Vitamins	Vitamin C, total ascorbic acid; niacin; pantothenic acid; vitamin B6; folate total; choline total	19.7 mg; 1.06 mg; 0.295 mg; 0.298 mg; 15 µg; 12.1 mg/100 g fw		
	Macro and micro elements	Calcium; magnesium; potassium; phosphorus; iron; zinc; manganese; copper; boron	9.40–11 mg ^11^; 16–28 mg; 280–580 mg; 59–60.57 mg; 0.65–1.49 mg; 0.26 mg; 0.15 mg; 0.095 mg; 0.10 mg/100 g fw		
Yacon	Polyphenolic compounds	Chlorogenic acid; caffeic acid; coumaric acid; total polyphenol content	Fractional concentration (%) of phenolic compounds: 53.72; 40.63; 4.38; 175.1 μg/g yacon syrup ^12^ 350–570 mg GAE and 790–3080 mg CAE/100 ^4^ g dw and 1202 μg GAE/g ^3^ yacon syrup	Antioxidant, anti-inflammatory, hypoglycemic, cardioprotective, prebiotic, anti-obesity	[95,96,97,98]
	Carotenoids	Carotene	80–130 μg/100 g fw		
	Vitamins	Thiamin; riboflavin; niacin, vitamin C	10–70 μg; 100–310 μg; 330 μg, 13 mg/100 g fw		
	Macro and micro elements	Phosphorus; potassium; calcium; magnesium; sulfur	23.2 mg; 171.7 mg; 6.3 mg; 3.7 mg; 9.7 mg/100 g fw yacon pulp		
Jerusalem artichoke	Polyphenolic compounds	Chlorogenic acid (CQA); dicaffeoyl isomers 3,5-diCQA caffeic acid; total phenols	1.48 mg; 0.82 mg; 70 μg/g dw; 7.4 mg GAE/g fw	Antioxidant, antibacterial, hepatoprotective, cardioprotective, anti-inflammatory, anti-obesity, anti-hypertension	[47,92]
	Carotenoids	β-Carotene	12 μg/100 g fw		
	Vitamins	Choline; niacin, vitamin C	30 mg; 1.3 mg; 4 mg		
	Macro and micro elements	Potassium; phosphorous; magnesium; calcium; sodium	490 mg; 78 mg; 17 mg; 14 mg; 4 mg/100 g fw		

^1^ Tocopherol equivalent; ^2^ dietary folate equivalent; ^3^ GAE—gallic acid equivalent; ^4^ CAE—caffeic acid equivalent ^5^ from white/purple potato to red/purple potato, ^6^ from yellow to red/purple potato, ^7^ from yellow to white/purple potato, ^8^ from white to red/purple potato, ^9^ red to purple potato, ^10^ white to dark yellow flesh, ^11^ potato without peel purple yam; ^12^—chlorogenic acid; fw—fresh weight; dw—dry weight; de—dry extract.

The antioxidant capacity strongly correlates with the concentration and profile of polyphenols and carotenoids. Betanin-rich beetroot and anthocyanin-rich sweet potatoes exhibit direct associations between these compounds and DPPH/ABTS values [33,64,65,66]. Processing such as baking or steaming can preserve or enhance antioxidant capacity by releasing bound compounds, although heat-sensitive vitamins like C may degrade [33,35,68]. Purple-flesh potatoes like ‘Vitelotte’ exhibited superior activity versus white varieties [92,93]. Taro leaves, rich in flavonoids, and Jerusalem artichoke, containing chlorogenic and caffeic acids, further support the contribution of these crops to antioxidative defense [33,47,64,65,66,67,68,69] (Table 2).

### 4.2. Glycemic Response

The glycemic index (GI) measures the postprandial effect of carbohydrates on blood glucose. Starch structure—particularly the amylose/amylopectin ratio—affects digestibility and GI. Amylose digests more slowly, increasing resistant starch (RS) formation, which acts as dietary fiber and improves glycemic control [99,100,101,102]. Polyphenols like chlorogenic and ferulic acids can inhibit α-amylase and α-glucosidase, slowing glucose absorption. Purple sweet potatoes, high in anthocyanins, exhibit stronger inhibitory effects and lower GI compared to white cultivars [68,70,103]. Carrots (GI: 35) and beetroot (GI: 64) enhance insulin sensitivity and reduce fasting glucose by 10–20 mg/dL in diabetic models [32,71,103].

In Monro et al.’s study [104], nine potato cultivars showed that RDS content dropped from 68% (fresh) to 44% (cold-stored), while RS increased from 3.9% to 7%, lowering GI significantly. Nayak et al. [102] confirmed that cooling boiled red potatoes reduced GI from over 70 to 56.2. Iancu [105] showed that adding cooked potatoes to bread raised RS content by 5.1%, improving glycemic outcomes. Sweet potatoes generally show GI values of 40–59 [106], while cassava flour substitutions reduced GI in bread to <50 with 100% replacement and cocoa addition [107]. Combining cassava, yam, taro, and sweet potato flours with rice flour lowered GI by 26% and increased RS content [107].

Jerusalem artichoke (JA), due to inulin content, reduced estimated GI of sourdough bread from 84.02 to 75.12 (with 20% JA powder) and also lowered GI in buckwheat extrudates by up to 42.3% [107,108,109]. Yacon, rich in fructooligosaccharides, lowered the GI of bread by 42% (with 11% flour addition), and its concentrate showed a GI of 40 [110]. Yam has a low GI of 51–66, and when used in bread, it raised RS content to ~42% [103,108,111]. Taro flour also reduced GI due to hydrolysis resistance, with values ranging from 55 to 77, depending on cultivar and processing [103,112,113].

## 5. Potentially Hazardous Compounds in Root and Tuber Vegetables

### 5.1. Acrylamide

Although root vegetables offer a plethora of benefits, including the potential for alternative processing methods and significant nutritional advantages, it is of equal importance to exercise caution regarding the potential formation of harmful compounds during high-temperature processing techniques [114]. One such compound is acrylamide (AA), which has been classified by the International Agency for Research on Cancer as “probably carcinogenic to humans”. As documented by the EFSA [115], AA is a chemical substance with documented neurotoxic, genotoxic, and carcinogenic properties. Its formation occurs in various foods during high-temperature treatments through the Maillard reaction, which involves the interaction between asparagine (Asn) and reducing sugars, primarily glucose and fructose, during processes such as frying, baking, and extrusion [114,115,116,117].

The content of asparagine in root vegetables, acting as a precursor in the release of AA in bakery products, varies significantly between species. Among root and tuber vegetables, potatoes exhibit the highest content of Asn, with an average range of 2030–4250 mg/kg fw [118]. The estimated potential for acrylamide formation in the analyzed potato samples ranged from 80 to 2020 µg/kg, with the levels of glucose and fructose exerting a significant influence. Despite their role as mediators, these compounds play an important part in the production of acrylamide [118]. With regard to the native variety of cassava designated as “530 cultivar”, the Asn content was determined to be 930 mg/kg. For diploid and tetraploid hybrids, the asparagine content was calculated based on the crude protein concentration and the percentage of asparagine in the total amino acids, with a range of 815.4 to 3976.18 mg/kg [119].

Research conducted by Lim et al. [120] shows that the highest amino acid concentration in sweet potatoes is glutamic acid, followed by asparagine and aspartic acid. The Asn content was 1976 mg/kg. The total content of asparagine and aspartic acid (Asx) residues in taro samples from the RIN (Ibo Ngaoundere) and CE (Country Ekona) varieties was measured at 144.3 mg/kg and 159.7 mg/kg for mucilage, and 152.3 mg/kg and 160.7 mg/kg for flour, respectively. These levels do not pose a risk for AA production in bakery products [121]. Studies conducted on juices obtained from two varieties of beetroots, “Wodan” and “Alto”, grown in Poland, showed that the Asn content was 489.02 and 797.17 mg/L respectively [122]. Similar values of AA precursor were obtained in fresh carrots; the Asn concentration was 520 mg/kg fw [123]. Low contents of Asn were shown in Jerusalem artichoke tubers. In the tested varieties, the Asn content ranged from 4.03 to 4.3 mg/kg and increased during 20 weeks of storage by 35–53% [124].

Varma’s study [125] found that different pre-treatments significantly reduced acrylamide formation in baked sweet potato, taro, and cassava. Garlic extract was the most effective, reducing levels by over 90%, while yeast and sodium chloride had minimal effects, varying by tuber type and treatment. Meanwhile, Sawicka et al. [126] demonstrated that incorporating potato flakes into bread formulations resulted in higher acrylamide levels in the crust, especially when larger quantities of potato were used in the flour blend. This finding underscores the importance of managing precursor compounds and considering other potential health risks associated with using tuber-based ingredients.

Also, Sawicka et al. [127] evaluated the effect of adding carrot strips on AA content in bread. Two carrot samples (Q1 and Q2) showed significant differences in Asn and AA levels. Q1 contained 91.1 mg/kg Asn and 35.7–38.3 μg/kg AA, with glucose and fructose at 445 and 25.7 mg/kg, respectively. Q2 had higher Asn (3554 mg/kg) but lower AA (16.6–17 μg/kg), with glucose at 15.8–16.4 mg/g. The addition of carrot significantly influenced AA content in bread. Control bread made with wheat flour and up to 20% added water had AA levels below 10 μg/kg. Bread with 22.5% Q2 and 10–20% water showed the highest AA levels (74–80 μg/kg). For Q1, adding 15–22.5% carrot at 0% water (quantity in control bread) increased AA to 62–73 μg/kg, while 10% water raised it to 50 μg/kg, meeting the EU benchmark. These results highlight the impact of carrot addition and water content on AA formation in bread.

According to Hamlet et al. [128], Asn concentrations varied significantly among cereals, with rye having the highest levels (396 mg/kg) and rice the lowest (61 mg/kg). In wheat, Asn concentrations ranged similarly in bread wheat flours, with white flours at 48–155 mg/kg and wholemeal flours at 106–346 mg/kg. The average Asn concentration in wholemeal wheat flour (255 mg/kg) was lower than in wholemeal bread flour (366 mg/kg), likely due to the lower protein content.

Acrylamide formation in baked products enriched with root and tuber crops is primarily determined by free asparagine, reducing sugar content, and thermal processing conditions. In bakery applications, mitigation strategies should focus on selecting low-asparagine cultivars, controlling sugar levels, and applying practical pre-processing methods such as blanching or soaking before drying. Optimizing moisture and baking parameters can further reduce acrylamide levels, supporting the safe use of root- and tuber-based flours without compromising technological performance or sensory properties.

### 5.2. Fermentable Oligo-, Di-, Monosaccharides, and Polyols (FODMAPs)

FODMAPs represent a class of short-chain carbohydrates that are poorly absorbed in the small intestine and highly fermentable by colonic microbiota. Their consumption may lead to gastrointestinal symptoms in sensitive individuals, particularly those with irritable bowel syndrome (IBS), due to osmotic activity and rapid microbial fermentation producing gas and short-chain fatty acids (SCFAs) [129]. Among plant-based foods, several root and tuber crops, especially those with high concentrations of fructans, mannitol, or galacto-oligosaccharides (GOS), are considered moderate to high FODMAP sources.

Notably, Jerusalem artichoke (*Helianthus tuberosus*) is one of the most concentrated natural sources of inulin-type fructans, with content ranging from 16% to 20% of dry weight, depending on cultivar, maturity stage, and postharvest storage conditions [3,130]. While this renders the crop highly valuable as a prebiotic and dietary fiber source, it also raises concerns regarding gastrointestinal tolerance in sensitive populations [131].

Yacon (*Smallanthus sonchifolius*), a South American tuber increasingly used in functional bakery applications, is similarly rich in fructooligosaccharides (FOS), comprising up to 40% of its dry matter content [132]. Although FOS intake has been associated with improvements in calcium absorption, lipid metabolism, and immune response [133], clinical studies also report dose-dependent gastrointestinal distress, including bloating, flatulence, and osmotic diarrhea, particularly at intakes exceeding 10–15 g/day [134].

Cassava (*Manihot esculenta*), widely consumed in tropical regions, typically contains lower levels of FODMAPs, though processing methods such as fermentation and soaking have been shown to further reduce residual oligosaccharides and improve digestibility [135]. Sweet potatoes (*Ipomoea batatas*), conversely, contain moderate amounts of mannitol and can elicit symptoms in some IBS patients, particularly when consumed in large portions (>100 g cooked weight) [136].

In contrast, potatoes (*Solanum tuberosum*) and taro (*Colocasia esculenta*) are generally classified as low-FODMAP tubers and are well tolerated by sensitive populations, making them suitable bases for functional bakery products aimed at consumers with FODMAP intolerance [137,138,139]. Notably, the majority of FODMAPs in tubers are water soluble and thus partially leach during boiling or blanching, emphasizing the importance of processing in modulating FODMAP levels [139]. From a technological perspective, high-FODMAP ingredients may present functionality advantages, such as enhanced water-binding, natural sweetness, and fat-replacement potential, due to their high solubility and fermentability. However, these benefits must be balanced against the risk of consumer intolerance. Controlled inclusion levels and proper labelling are critical for product design targeting sensitive individuals [140].

The fermentation of FODMAP-rich tuber flours (e.g., from Jerusalem artichoke or yacon) with selected lactic acid bacteria can reduce oligosaccharide content and enhance postbiotic generation, including SCFAs and bacteriocins, potentially offsetting their adverse effects [141]. Similarly, enzymatic hydrolysis using inulinases or invertases has been proposed as a tool to selectively degrade problematic oligosaccharides prior to baking [142]. The FODMAP content of edible tubers is highly variable and species dependent, with Jerusalem artichoke and yacon presenting the highest levels, while potatoes, taro, and cassava generally remain within low-FODMAP thresholds. Given the increasing prevalence of functional gastrointestinal disorders and consumer demand for sensitive-friendly bakery products, the characterization and modulation of FODMAP content is of considerable nutritional and industrial importance.

Elevated levels of FODMAPs derived from certain tuber-based flours may reduce gastrointestinal tolerance in gluten-free, conventional, and fiber-enriched baked goods, particularly among sensitive individuals. Although gluten-free products are often perceived as more easily digestible, a recent comparative analysis demonstrated that not all such products are low in FODMAPs; their content largely depends on the type and proportion of raw materials used [143]. This is particularly relevant for gluten-free formulations, where high-FODMAP tubers such as Jerusalem artichoke and yacon are commonly incorporated to enhance texture, moisture retention, and nutritional quality. Consequently, formulation strategies such as sourdough fermentation, enzymatic degradation, or portion size control are recommended to mitigate these effects and improve gastrointestinal tolerance [137].

### 5.3. Phytate

Phytate is an organic anion derived from phytic acid (inositol hexakisphosphate). It occurs in many plants, including root vegetables. It reduces mineral bioavailability by forming insoluble complexes with calcium, iron, zinc, and magnesium and inhibits digestive enzymes such as trypsin and amylase. Despite its antinutritional effects, phytate offers health benefits, including antioxidant activity, cholesterol reduction, cancer protection, and blood glucose regulation. Thermal processing, such as boiling, can lower phytate content by around 20%, improving mineral absorption [88,144].

Phytate levels in plant foods differ depending on the plant variety and specific plant parts, influenced by their unique genetic and physiological characteristics. The phytate content in selected root vegetables and tubers is presented in Table 3 [145,146].

Phytate levels can be reduced through technological processing during food preparation. Studies have shown that boiling for 15 min decreased phytate content in beetroot and carrots by 52 mg/100 g and 26 mg/100 g, respectively, corresponding to respective reductions of 22.4% and 46.9% [145]. Research investigating the impact of heat treatment on phytate content in potatoes demonstrated that cooking and microwave cooking reduced phytate levels by 15% and 25%, respectively. However, baking did not result in significant differences compared to raw potatoes. Similar research by Bhandari and Kawabata [156] analyzed four yam varieties with phytate levels ranging from 46 to 72 mg/100 gfw. Cooking was identified as the most effective heat treatment, reducing phytate content by an average of 22%, while pressure cooking and baking resulted in slight reductions of 6% and 3%, respectively [157]. Ayele et al. [88] demonstrated that controlled cooking at 91 °C reduced phytate content by 24% in yam and 26% in taro. Research on the incorporation of cassava flour (CF) in wheat bread production demonstrated that increasing the share of CF as a replacement for wheat flour significantly reduced phytate contents, with reductions ranging from 19.95% at 10% CF to 34.31% at 100% CF. The reference sample, made from wheat flour, had a phytate content of 15.04 mg/g [108]. Asiyanbi and Hammed [163], in their analysis comparing the physicochemical properties of wheat flour (WF) and fermented (F) and unfermented yam flour (UYF), found that yam flours had significantly lower phytic acid content, with values of 17 mg/100 g and 53 mg/100 g, respectively, compared to wheat flour (225 mg/100 g). Fermentation of yam flour notably reduced phytic acid content by 92% in WF and 68% in UYF.

### 5.4. Oxalate

Oxalate is the anionic form of oxalic acid, a simple dicarboxylic acid naturally occurring in various organisms, from microorganisms to plants and animals. In the human body, oxalate can originate not only from dietary sources but also from the metabolism of ascorbic acid and glyoxylate [164]. Excessive intake of oxalates may lead to oxalosis and the deposition of calcium oxalate in body tissues and organs [165]. In individuals with kidney stone disease, it is recommended to limit oxalate intake to less than 40–50 mg per day [166], which highlights the importance of determining oxalate content in food. Oxalates are naturally occurring antinutritional compounds commonly found in root and tuber vegetables. Their presence can affect the bioavailability of essential minerals such as calcium, iron, and magnesium, and at elevated concentrations, they may promote the formation of kidney stones. The oxalate content is presented in Table 3. In the case of carrots (*Daucus carota*), the oxalate content was reported to be 5 mg/100 g at concentrations low enough not to pose any risk to consumer health [84]. For beetroot juice (*Beta vulgaris*), the oxalate content ranged from 54 to 60 mg/100 g [156,157].

Substantial variation in oxalate content has been observed in yam tubers, with levels ranging from 67 to 197 mg/100 g fw in raw samples. Thermal processing, such as boiling, reduced oxalate content to between 31 and 106 mg/100 g fw, corresponding to an average reduction of 39%. The least effective method was baking, which resulted in only an 11% reduction [156]. In Jerusalem artichoke (*Helianthus tuberosus*), oxalate levels ranged from 0.9 to 22 mg/100 g fw [161]. Taro flour contained 26.31 mg/100 g of oxalate [152], whereas analyses of various taro genotypes revealed a wide variation in oxalate content, ranging from 372.89 to 2386.55 mg/100 g fw [167].

### 5.5. Cyanogenic Glycosides

Cyanogenic glycosides are natural plant products derived from secondary metabolism. These compounds consist of an α-hydroxynitrile aglycone and a sugar component, typically D-glucose. These compounds, primarily linamarin and lotaustralin, are nitrogen-containing secondary metabolites produced by the plant as a defense mechanism. They consist of an α-hydroxynitrile aglycone and a sugar component, typically D-glucose [168,169].

When consumed without proper processing, these glycosides are hydrolyzed by the bacterial microflora of the gastrointestinal tract to produce hydrogen cyanide (HCN), a toxic compound that can cause acute or chronic health effects. At high oral doses, symptoms appear within minutes and may include nausea, vomiting, dizziness, headache, heart palpitations, rapid breathing followed by difficulty breathing, slowed heart rate, unconsciousness, and convulsions, ultimately leading to death [169]. The minimal lethal dose of cyanide in humans is frequently cited as approximately 0.5 mg/kg of body weight. Chronic exposure to lower doses of cyanide can result in neurological disorders, particularly in populations that rely on cassava as a dietary staple in tropical regions [170]. The detoxification process entails the metabolism of cyanide, primarily by the mitochondrial enzyme sulfur transferase (TST, rhodanese). This enzyme facilitates the conversion of cyanide into the less toxic thiocyanate, which is subsequently excreted in urine. The efficiency of this process is contingent upon the availability of sulfur compounds, which serve as a limiting factor [171].

Among root vegetables, cassava is notable for its relatively high content of cyanogenic glycosides, which can release hydrogen cyanide (HCN) upon enzymatic hydrolysis. According to available data, the total cyanogenic potential (expressed as HCN equivalent) ranges from 53 to 1300 mg/kg dw in the leaves and from 10 to 500 mg/kg dw in the roots [172,173]. This variability is also reflected in cassava flours, where the residual HCN content depends mainly on the cultivar and processing methods. For example, in Sri Lankan varieties, HCN levels ranged from 4.85 to 48.05 mg/kg, with some exceeding the WHO-recommended limit of 10 mg/kg for safe consumption [174]. Traditionally, soaked cassava chips and roots contain relatively high HCN levels (46.6 and 60.98 mg/kg, respectively), while cassava paste/dough averages 38.1 mg/kg. In contrast, processed products such as cassava biscuits (12 mg/kg) and especially gari, a fermented and roasted product (5.7 mg/kg), exhibit markedly lower HCN concentrations [175]. Those results prove that thermal processing alone is insufficient to eliminate cyanogenic compounds and that detoxification techniques—such as fermentation, soaking, and drying—are essential before incorporating cassava flour into bakery formulations.

Despite the high concentration of cyanide in cassava roots, which form a staple component of the diet in many countries, its content can be significantly reduced through the application of the following technological processes:

Peeling—Removing the peel reduces the cyanogenic glycoside content by approximately 50%. This method is particularly effective for sweet cassava varieties [176].

Sun drying of grating/slicing (<5 mm)–Mechanical fragmentation of cassava increases the surface area for interaction between cyanogenic glycosides and the enzyme linamarase, facilitating the breakdown and release of volatile hydrogen cyanide (HCN). Drying (14 h) detoxification is influenced by factors such as moisture content, the rate of moisture removal, and potentially enzyme activity. This process has the potential to reduce cyanogenic compounds by up to 65.9% (G) depending on the degree of fragmentation [177].

Soaking prior to sun drying—The process of soaking cassava in water for a period of 24 h induces hydrolysis of cyanogenic glycosides, potentially reducing HCN levels by up to 90% [178].

Fermentation—Fermentation using specific microorganisms, such as *Aspergillus niger* or lactic acid bacteria (*Lactobacillus plantarum*), is highly effective. It can lower HCN content by up to 95% while increasing cassava protein content by 50% [179].

Cooking and baking—The boiling of cassava in water inactivates enzymes that degrade cyanogenic glycosides, thereby reducing toxins. The reduction is more pronounced when smaller pieces of cassava (75% HCN, 2 g) are used in comparison to larger chunks (25% HCN, 50 g). Baking at 110 °C for 20 min reduces cyanide by 13% [23,180]. In baking, cassava flour must be properly detoxified through fermentation, soaking, and drying before use, as baking alone does not eliminate cyanogenic compounds. This is crucial for traditional dishes like cassabe, a South American flatbread made from soaked and fermented cassava or freshly grated roots, with detoxification depending on the variety and cyanide content [23]. Cassava’s gluten-free nature makes it attractive for functional and allergen-free bakery formulations; however, its use must be carefully controlled to ensure both safety—due to cyanogenic glycosides—and adequate product quality.

### 5.6. Steroidal Glycoalkaloids

α-Solanine and α-chaconine are the principal steroidal glycoalkaloids (SGAs) found in potato tubers, accounting for approximately 95% of the total glycoalkaloid content (TGA). While these compounds contribute to the plant’s defense mechanisms, they can be toxic to humans when consumed in high amounts [181]. SGAs exhibit beneficial bioactive properties at low concentrations, such as anti-inflammatory, anticancer, and cholesterol-lowering effects. However, excessive intake may lead to toxicity. Clinical symptoms can occur after consuming 1–5 mg TGA/kg body weight, with severe poisoning or death possible at 3–6 mg/kg. Reported symptoms include gastrointestinal (nausea, vomiting, abdominal pain, diarrhea), neurological (dizziness, drowsiness, visual disturbances, seizures), and, in rarer cases, hypothermia, tachycardia, respiratory failure, or coma [182,183]. Regulatory authorities recommend that commercial potato cultivars’ TGA levels not exceed 200 mg/kg fresh weight [181].

Commercial mashed potato flakes show notable variation in glycoalkaloid content, primarily influenced by raw material selection and processing. In a comparative analysis of six commercial products, TGA levels ranged from 33.86 to 81.59 mg/kg, with higher concentrations associated with products likely containing more potato peel, where glycoalkaloids are most concentrated. In contrast, peeled Idaho potatoes used as a control contained only 19.92 mg/kg. The predominance of α-chaconine in several products supports the hypothesis of limited peel removal during processing [184].

Given the tight safety margins and variability in human sensitivity, targeted interventions are essential to mitigate glycoalkaloid content in potato-derived food matrices. The following strategies represent a comprehensive, multi-level approach to reducing TGA levels across the potato supply chain:

Peeling and blanching—Glycoalkaloids are primarily concentrated in the potato peel and the outer 1–1.5 mm of the tuber cortex. Thus, peeling alone can remove up to 60% of total TGA content, while peeling followed by blanching of the tubers in hot water can further increase the reduction up to 90%, due to enhanced leaching and enzymatic deactivation [185,186].

Acidic soaking—Short-term soaking of blanched potato slices in 1% organic acid solutions (e.g., malic, citric, or lactic acid) significantly reduces glycoalkaloid levels—up to 97% for α-chaconine in snacks and 93% in French fries, especially in colored-flesh varieties [187].

Thermal processing—Thermal methods significantly reduced TGA content in potato peels by 52–84%, with frying, baking, and air frying at high temperatures being the most effective, achieving reductions of over 75%, while boiling and steaming were less effective [188]. In whole potatoes, glycoalkaloid levels decrease by approximately 20% after peeling, 8–39% after boiling, and up to 94% after frying, with greater reductions observed in fries than in chips. Boiling has also been more effective than microwaving, resulting in a 40–50% reduction in TGA content [181]. Drying is likewise highly effective, with studies reporting total glycoalkaloid reductions of approximately 80% when preceded by boiling [189].

Cultivar selection—Glycoalkaloid levels vary by potato variety. Colored-flesh cultivars like Double Fun and Mulberry Beauty often have lower TGA content [187]. The selection or breeding of cultivars with low glycoalkaloid content represents a key long-term strategy to reduce dietary exposure to these toxic compounds.

Biotechnological and microbial approaches—Emerging research suggests that certain microbes, such as *Arthrobacter* spp., and edible fungi like *Pleurotus pulmonarius* can enzymatically degrade glycoalkaloids to non-toxic metabolites such as solanidine. Although these methods are still experimental, they offer future promise for processing waste streams and valorizing potato pulp [190].

The use of potato-based flours or dried potato products in bakery applications may pose a safety risk due to the thermal stability of glycoalkaloids. α-Solanine and α-chaconine are not significantly degraded during baking (decomposition temperature above 170 °C), and their levels may persist in the final product, mainly if derived from unpeeled, inadequately processed, or improperly stored tubers. Therefore, a quality control strategy, including peeling, blanching (optional), and raw material selection, is essential to ensure the safety of potato-enriched breads and pastries.

A comparison of anti-nutritional risks in tubers is summarized in Table 4.

## 6. Conclusions

Root and tuber crops are valuable sources of bioactive compounds, offering significant health benefits and functional properties for bakery product development. This review demonstrates that various species, including potato, cassava, beetroot, carrot, yam, and sweet potato, provide compounds such as polyphenols, resistant starch, carotenoids, vitamins, minerals, and prebiotic fibers, which may enhance antioxidant potential, modulate glycemic response, and improve the nutritional profile of baked goods.

However, several limitations and risks must be considered. The presence of acrylamide, formed during high-temperature baking of starchy tubers, poses a potential health risk. Additionally, cyanogenic glycosides in cassava and high FODMAP content in Jerusalem artichoke and yacon may lead to gastrointestinal discomfort or toxicity if not properly processed. These safety concerns underscore the need for adequate technological treatments and standardization protocols.

Despite their promising potential, consumer acceptance may be hindered by color, taste, or texture changes when substituting traditional flour with root vegetable flours. Further research on sensory optimization, labelling, and education is needed to bridge the gap between nutritional value and market viability.

From a practical standpoint, industry stakeholders should focus on optimizing processing methods (e.g., steam blanching, fermentation, low-temperature baking) to enhance the stability and bioavailability of target compounds while minimizing harmful by-products. Nutritionists and food technologists are encouraged to formulate multi-functional bakery products using well-characterized tuber-based ingredients tailored for populations with metabolic disorders, such as diabetes or obesity.

While root and tuber crops offer notable benefits for health-oriented baking, their successful integration into food systems requires balancing functionality, safety, and consumer preferences. A multi-disciplinary approach involving food science, nutrition, and consumer research is essential to unlock their full potential and mitigate the associated drawbacks. A limitation of this study is the absence of a meta-analysis and the exclusion of non-English articles, which may have restricted the breadth of included studies.

## Figures and Tables

**Table 1:** Chemical composition of root vegetables per 100 g of fresh weight (%).

Raw Material	Energy [kcal/100 g]	Ash [%]	Carbohydrates [%]	Simple Sugars [%]	FOS [%]	Fat [%]	Protein [%]	Dietary Fiber [%]	Water [%]	References
Carrot	41	0.7–4.5	7–10	F: 0.8; G: 0.4; S: 4.5	0.1	0.1	0.9	1.2	86–89	[8,9,10]
Potatoes	80	2.1	16.8	F: 0.9; G: 1.0; S: 1.5	ND	0.25	1.8	1.30	79	[9,11,12]
Sweet potatoes	79	1.2	17.3	F: 0.1.2; G: 0.1–1.2; S: 1.6–2.6	0.1–0.2	0.2	2.0	4.6	63.3–94.4	[9,13,14]
Beetroot	43	1.1	9.6	Total 6.76	ND	0.17	1.6	2.8	87.6	[9,15,16]
Taro	112	1.2	26.4	F: 0.2–1.4; G: 0.4; S: 0.8–2.3	0.1	0.2	1.5	2.45–4.1	70.64	[9,17,18]
Yam	118	1.0–3.8	17.9–21.9	F: 0.2–1.4; G: 0.14–2.0; S: 0.4–0.7	0.2	0.2 –0.5	3.7–7.5	4.1	68.1–73.3	[9,19,20]
Cassava	110–160	0.4–1.7	25.3–38.1	F: 0.3; G: 0.2; S: 1.7	0.1–0.2	0.1–0.3	0.3–3.5	0.1–3.7	59.7	[9,21,22,23]
Yacon	46–56	0.2–0.3	10.6–13.1	F: 0.9; G: 0.4; S: 1.4	4–9.1	0.1–0.2	0.37–0.7	0.4–1.1	85.9–86.8	[20,24,25,26]
Jerusalem artichoke	73	1.9–2.5	15.8–17.4	Total 9.6	12–15	0.1	1.8–2	1.6	80.3	[27,28]

G—glucose, F—fructose, S—sucrose, FOS—fructooligosaccharides, ND—not detected.

**Table 3:** Oxalate and phytate content ranges (mg/100 g fw) by vegetable tubers.

Family	Species	Oxalate	Phytate	References ^1^	References ^2^
Apiaceae	Carrot (*Daucus carota*)	3–49	15–88; 446 **	[147,148]	[149,150]
Amaranthaceae	Beetroot (*Beta vulgaris* L. Var. Vulgaris)	39–109	5–52	[147,151]	[147,152]
Araceae	Taro (*Colocasia esculenta* L. Schott)	26.3–109	49.6–169	[153,154,155]	[88,152]
Solanaceae	Potato (*Solanum tuberosum* L.)	ND–26	21–55	[156]	[157]
Dioscoreaceae	Yam (*Discorea deltoidea*)	4–197	46–72; 184–363 *; 637	[158]	[94,152,158]
Convolvulaceae	Sweet potato (*Ipomoea batatas* L. Lam.)	467.3–523.9 **; 25.6–793.3 *	ND–12; 50–420 **	[145,159,160]	[145,157]
Euphorbiaceae	Cassava (*Manihot esculenta*)	15.7	191.2–624	[62]	[63,146]
Asteraceae	Jerusalem artichoke *Helianthus tuberosus*	0.9–22	30–190	[161]	[152,161]
	Yacon (*Smallanthus sonchifolius*)	8.5; 68.1 **	273	[96]	[162]

* Dry basis, ** dry mass basis, ND—not detected; ^1^—references for oxalates; ^2^—references for phytates.

**Table 4:** Comparative table of anti-nutritional risks in tubers.

Tuber	AA	HCN	α-Solanine, α-Chaconine	Phytate	Oxalate	FODMAPs
Cassava	Moderate-High	Moderate-High	N.d./N	Moderate-High	Low	Low
Potato	Moderate-High	N.d./N	Moderate-High	Low	Low	Low
Sweet potato	Moderate	N.d./N	N.d./N	Low	Moderate-High	Low
Beetroot	Low-Moderate	N.d./N	N.d./N	Low	Moderate	Low
Carrot	Low	N.d./N	N.d./N	Low	Low	Low
Taro	Low	N.d./N	N.d./N	Moderate	Moderate	Low
Yam	Low	N.d./N	N.d./N	Moderate	Moderate	Low
Jerusalem artichoke	Low	N.d./N	N.d./N	Low-Moderate	Low	High (inulin rich)
Yacon	Low	N.d./N	N.d./N	Moderate	Low	High (FOS rich)

N.d./N—Not detected or not reported in the reviewed literature.
